# Preoperative pain measurements in correlation to deep endometriosis classification with Enzian

**DOI:** 10.52054/FVVO.14.3.034

**Published:** 2022-09-30

**Authors:** J Metzemaekers, M.D. Blikkendaal, K.E. V. Nieuwenhuizen, K Bronsgeest, J.P.T. Rhemrev, M.J.G.H. Smeets, J English, F.W. Jansen, S Both, A.R.H. Twijnstra

**Affiliations:** Department of Gynaecology, Leiden University Medical Center, Leiden, the Netherlands; Department of Gynaecology, Haaglanden Medisch Centrum-Bronovo, Den Haag, the Netherlands; Department of Biomechanical Engineering, Delft University of Technology, Delft, the Netherlands; Department of Sexology & Psychosomatic Gynecology, Amsterdam UMC, Amsterdam, the Netherlands

**Keywords:** Endometriosis, surgery, rASRM, Enzian, classification

## Abstract

**Background:**

Deep Endometriosis (DE) classification studies with Enzian never compared solitary compartments (A, B, C, F), and combinations of anatomical locations (A&B, A&C, B&C, A&B&C), in correlation to pain. Therefore, the results of these studies are challenging to translate to the clinical situation.

**Objectives:**

We studied pain symptoms and their correlation with the solitary and combinations of anatomical locations of deep endometriosis lesion(s) classified by the Enzian score.

**Materials and Methods:**

A prospective multi-centre study was conducted with data from university and non- university hospitals. A total of 419 surgical DE cases were collected with the web-based application called EQUSUM (www.equsum.org).

**Main outcome measures:**

Preoperative reported numeric rating scale (NRS) were collected along with the Enzian classification. Baseline characteristics, pain scores, surgical procedure and extent of the disease were also collected.

**Results:**

In general, more extensive involvement of DE does not lead to an increase in the numerical rating scale for pain measures. However, dysuria and bladder involvement do show a clear correlation AUC 0.62 (SE 0.04, CI 0.54-0.71, p< 0.01). Regarding the predictive value of dyschezia, we found a weak, but significant correlation with ureteric involvement; AUC 0.60 (SE 0.04, CI 0.53-0.67, p< 0.01).

**Conclusions:**

Pain symptoms poorly correlate with anatomical locations of deep endometriosis in almost all pain scores, with the exception of bladder involvement and dysuria which did show a correlation. Also, dyschezia seems to have predictive value for DE ureteric involvement and therefore MRI or ultrasound imaging (ureter and kidney) could be recommended in the preoperative workup of these patients.

**What's new?:**

Dyschezia might have a predictive value in detecting ureteric involvement.

## Introduction

An ideal classification system should meet three core principles: it should be simple, allow description of the disease and correlate with clinical findings (Adamson, 2011). Currently, the two most used classifications systems for endometriosis are the rASRM (revised American Society for Reproductive Medicine, 1997) and Enzian (Keckstein et al., 2003), of which neither shows strong clinical correlation with pain symptoms (Haas et al., 2013a; Montanari et al., 2019). This could be explained by the fact that the rASRM is not directly suitable to describe deep endometriosis (Haas et al., 2013b). While on the other hand, the Enzian classification is not able to describe the tubes, ovaries and peritoneum which is covered by the rASRM classification. An attempt to overcome these difficulties has recently been made by the Stiftung Endometriose Forschung (SEF) at their annual Weisensee endometriosis meeting, during which an update of the Enzian classification was created: the #Enzian (Keckstein et al., 2021).

Previous studies (Haas et al., 2013a; Montanari et al., 2019) reported correlations between Enzian and pain symptoms in deep endometriosis. Haas et al. (2013a) reported no combinations of compartments but choose to report the compartment with the highest severity: for example, A1B0C3 would become a solitary C in the analysis. In real life, endometriosis does not respect the compartment boundaries of the Enzian classification and therefore it is difficult to translate these findings directly to the clinical symptoms. Montanari et al. (2019) performed a similar analysis with a more robust cohort. Nevertheless, both studies failed to study the solitary compartments and combinations of compartments in correlation to pain symptoms.

Therefore, the research questions that we addressed were: 1) Which compartment is responsible for the severity of the different types of pain? 2) Does pain increase with an increase in compartment involvement? 3) Do specific clinical symptoms correlate with the scoring in the Enzian?

The primary aim of this study was to find a correlation of the Enzian score with pain symptoms in deep endometriosis (DE). Secondly, we wished to determine whether a correlation exists between the extent of the disease and the reported intensity of pain (regarding solitary compartments and combinations of compartments).

## Materials and methods

### Study design

We performed a multi-centre study (university hospitals, non-university hospitals and specialised clinics for endometriosis). Surgical DE cases were prospectively collected from eleven centres in six European countries, of which the majority of inclusions were from the Netherlands (five centres), Finland (one centre), France (two centres), Germany (one centre), Switzerland (one centre), Romania (one centre), with a median inclusion rate of 8.5 (IQR 1-49) cases.

### Data collection

Data was collected from February 2019 through June 2020 with the EQUSUM application. Registered data included: general data on patients’ characteristics, previous abdominal surgery, pain scores for dysmenorrhoea, dyschezia, dysuria, chronic pelvic pain, dyspareunia, accurate localisation of endometriosis lesions, and in case of fertility wish, the EFI (endometriosis fertility index) scores (Adamson and Pasta, 2010). The EQUSUM application automatically generates the following classifications: rASRM, Enzian and EFI scores. For this research question only the Enzian classification is studied. The surgeon/gynaecologist filled in the surgical procedure on the EQUSUM application.

The classification is based on the surgical findings and not on the radiological or pathological findings. Pain scores were preoperatively documented with a numeric rating scale (NRS) ranging from 0-10. The Enzian scores were assigned according to the original manuscript (Keckstein et al., 2003).

Ethical approval was given by the Medical Ethics Committee of the Leiden University Medical Centre (LUMC) (G20.019).

The inclusion criterion was the presence of deep endometriosis in one or more of the main Enzian compartments A, B, C or F. Deep endometriosis was staged on the clinical intra-operative view combined with preoperative imaging. This routine work-up included the use of ultrasound and MRI. The surgical findings were leading in the EQUSUM classification.

Exclusion criteria were procedures which showed no disease involvement in one of the main compartments (A, B, C or F).

### Definition of DE

For this study we used the definition of DE described by the international working group of AAGL, ESGE, ESHRE and WES in 2021 (Tomassetti et al., 2021), which we classified with the Enzian based on clinical expertise combined with radiological and intra-operative findings. The definition for DE is: an endometrium-like tissue lesions in the abdomen, extending on or under the peritoneal surface. Usually nodular, able to invade adjacent structures, and associated with fibrosis and disruption of normal anatomy.

### Compartment involvement

Pain scores were calculated for all main compartments, so the solitary groups A, B and C without involvement of other compartment. This implies that for example only options of A1/2/3 B0, C0, Fx would be assigned to compartment A. Combinations were also possible, for example A1B2C0, would result in the group AB and so on. The assignment to the main compartments was irrespective of the involvement of F. Solitary mean pain calculations for the compartments F (FB, FA, FU and FI) were calculated with no involvement of any main compartments (e.g., A0B0C0, FB). This was performed to present the pain scores with the least bias of other compartments. To present the overlay of certain compartments we used euler diagrams.

To test if endometriosis pain symptoms could predict deep endometriosis involvement, we calculated receiver operating characteristic (ROC) curves. The test in this case is the presence of endometriosis classified with the Enzian (involvement yes or no), and the continuous variable is the severity of symptoms measured with the NRS scale (0-10).

### Statistical analyses

IBM SPSS version 25.0 for Windows was used for our analysis and we used the Shapiro–Wilk test to evaluate the distribution of the data. Data are presented as mean ± standard deviation (SD) or median (with interquartile range) for normally distributed or skewed data, respectively. Euler eclipse diagrams were created with online software based on an R package called euler. To test the accuracy of symptoms in correlation to the anatomical location, ROC curves and the areas under the curves were obtained for each anatomical location. We considered a 2-tailed p-value of < 0.05 as statistically significant. And for the ROC curve we considered an area under the curve of ≥0.6 as a discriminating test (Hosmer Jr et al., 2013).

## Results

In total 475 procedures were registered. Nine cases were removed because of diagnostic procedures and 47 cases were removed because no deep endometriosis was present in compartment A, B, C or F. This resulted in a total of 419 DE cases. Baseline characteristics are presented in Table I. The mean age of women who underwent surgery was 35.3±6.5 years, with a median Body Mass Index (BMI) of 24 (IQR 21-28) kg/m2. The majority of women had one or more previous abdominal surgeries; 13% reported more than 2 procedures (endometriosis and non- endometriosis surgeries). The primary indication for surgery were pain symptoms (82%).

**Table I:** Baseline Measures

Total procedures		N= 419
Age mean, SD		35.3±6.5
BMI median, Q1-Q3		24 (21-28)
Previous abdominal surgery	0	167 (39.9%)
	1	139 (33.2%)
	2	57 (13.6%)
	>2	56 (13.4%)
Surgery type	Laparotomy	1 (0.2%)
	Laparoscopic	416 (99.3%)
	Robotic	2 (0.5%)
Primary indication for surgery	Pain	342 (81.6%)
	Fertility	67 (16.0%)
	Cyst formation	3 (0.7%)
	Organ damage*	5 (1.2%)
	Abnormal uterine bleeding	1 (0.2%)
	Other	1 (0.2%)

*e.g., obstruction of ureter

Table II shows all classification types. Regarding the Enzian classification, compartment A was involved in 110 cases (26.3%), with a majority being A3 (55.5%). Compartment B was reported in 244 cases (58.2%) on the left side and 229 cases (52.3%) on the right side. The majority had a B2 severity (50.4% left and 53.7% right). Sacro-uterine involvement was reported the most, and cardinal ligaments the least. Compartment C was reported in 189 cases (45.1%), with the majority having a C3 severity (55.6%). Adenomyosis (FA) was reported in 58% of all cases, with most of the diffuse type (62.6%). Bladder (FB) involvement was reported in 71 cases (16.9%). Intestinal involvement (FI) was reported in 94 cases (22.4%), with 49.6% rectum involvement cranial to the recto-sigmoid junction, appendix involvement in 33.6%, ileocaecal in 7.6%, caecum in 6.7% and ileum in 2.5% of the FI cases. Ureteric involvement (FU left) was reported in 60 cases (14.3%), with hydronephrosis in 6 cases (10%). FU on the right side was reported in 44 cases (10.5%), with 6 cases hydronephrosis (13.6%). Other Enzian involvement (FO) was reported in 23 cases (5.5%), with 95.6% umbilical and 4.3% diaphragmatic lesions.

**Table II:** Enzian distribution

Enzian		1(<1 cm)	2(1-3 cm)	3(>3 cm)
A (rectovaginal space, vagina)	110 (26.3%)	12 (10.9%)	37 (33.6%)	61 (55.5%)
B left (uterosacral ligaments)	244 (58.2%)	51 (20.9%)	123 (50.4%)	70 (28.7%)
-Cardinal	2.10%			
-External ureter compression	5.20%			
-Sacrouterine	54.40%			
-Sacrouterine and cardinal	19.90%			
-Pelvic side wall	18.30%			
B right (uterosacral ligaments)	229 (52.3%)	47 (20.5%)	123 (53.7%)	59 (25.8%)
-Cardinal	2.70%			
-External ureter compression	3.70%			
-Sacrouterine	55.10%			
-Sacrouterine and cardinal	20.60%			
-Pelvic side wall	17.90%			
C (rectum)	189 (45.1%)	13 (6.9%)	71 (37.6%)	105 (55.6%)
FA (adenomyosis)	243 (58%)			
-Focal (well-defined mass in the myometrium)	29.60%			
Difusse (heterogeneity in myometrial aspect)	62.60%			
-Adenomyoma	7.00%			
-Other	0.80%			
FB (bladder)	71 (16.9%)			
FI (intestine)	94 (22.4%)			
-Rectum cranial to sig junction	49.60%			
-Coecum	6.70%			
-Ileocoecaal	7.60%			
-Ileum	2.50%			
-Appendix	33.60%			
FU left (ureter)	60 (14.3%)			
-hydronefrosis	6 (10%)			
FU right (ureter)	44 (10.5%)			
-hydronefrosis	6 (13.6%)			
FO (other locations)	23 (5.5%)			
-Diaphragm	4.30%			
-Umbilicus	95.60%			

## Preoperative numeric pain scale

Table III shows the median pain scores, ranging from most painful (dysmenorrhoea) to least painful (dysuria). For dysmenorrhoea (NRS 0-10) a median of 8 (IQR 7-9) was reported. A mean dyschezia of 4.9±3.7, dyspareunia 4.5±3.5, chronic pelvic pain (CPP) 4.5±3.5 and dysuria symptoms with a mean pain score of 2.1±3.0.

**Table III:** Numeric rating scores for pain (0-10).

	n=391
Pain symptoms	NRS
dysmenorrhoea , median (IQR)	8 (7-9)*
dyschezia, mean (SD)	4.9±3.7
CCP, mean (SD)	4.5±3.5
dyspareunia, mean (SD)	4.5±3.5
dysuria, mean (SD)	2.1±3.0

*n=375, non-normal distribution of the data therefore median, the other pain scales were normal distributed using the mean.

**Figure 1:**
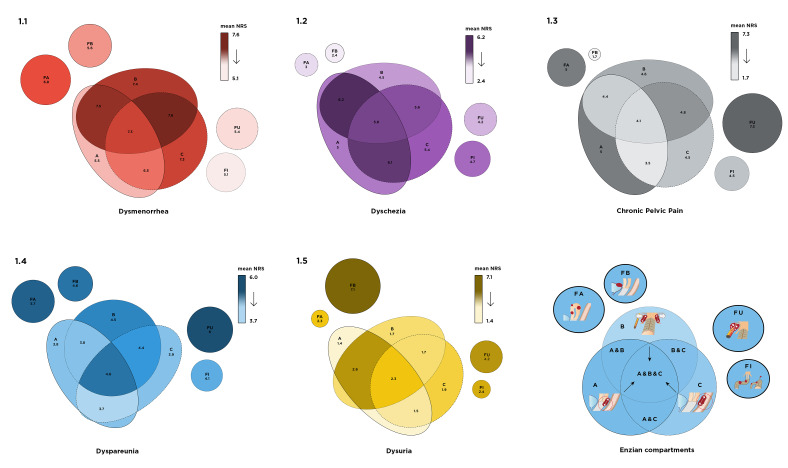
Schematic euler diagrams of solitary and combined Enzian compartments. Darker color gradient means higher pain score.

Solitary and grouped pain scores (0-10) for the different Enzian compartments are presented in Figure 1.

### Dysmenorrhoea Figure 1.1 (red)

A mean pain for compartment A was found of 5.5, SE 2.10 (n=6), B 7.4, SE 0.27 (n=125), C 7.3, SE 0.54 (n=34), A&B 7.5 SE 0.62 (n=24), A&C 6.5 SE 0.68 (n=19), B&C 7.6 SE 0.33(n=75) and A&B&C 7.3 SE 0.39 (n=61). For the F compartments: FB 5.6 SE 1.42 (n=10), FA 6.8 SE 0.47 (n=63), FI 5.1 SE 1.15 (n=15), FU 5.4 SE 2.27 (n=6). No dysmenorrhoea but with Enzian involvement is present in Supporting Information Table SI n=1 (16.7%) in compartment A, n=10 (8%) in compartment B, n=3 (8.8%) in compartment C, n=3 (30%) in compartment FB, n=11(17.5%) in compartment FA, n=4 (26.7%) in compartment FI and n=2 (33.3%) in FU.

### Dyschezia Figure 1.2 (purple)

A mean pain for compartment A was found of 5.0, SE 2.05 (n=6), B 4.5, SE 0.36 (n=125), C 5.4, SE 0.63 (n=34), A&B 6.2 SE 0.73 (n=24), A&C 6.1 SE 0.76 (n=19), B&C 5.6 SE 0.41 (n=75) and A&B&C 5.8 SE 0.43 (n=61). For the F compartments: FB 2.4 SE 1.25 (n=10), FA 3.0 SE 0.46 (n=63), FI 4.7 SE 1.12 (n=15), FU 4.2 SE 1.90 (n=6). No dyschezia but with Enzian involvement is present in Supporting Information Table SI n=2 (33.3%) in compartment A, n=40 (32.0%) in compartment B, n=8 (23.5%) in compartment C, n=7 (70.0%) in compartment FB, n=31 (49.2%) in compartment FA, n=5 (33.3%) in compartment FI and n=3 (50.0%) in FU.

### Chronic pelvic pain Figure 1.3 (grey)

A mean pain for compartment A was found of 5.0, SE 1.10 (n=6), B 4.6, SE 0.33 (n=125), C 4.5, SE 0.59 (n=34), A&B 4.4 SE 0.79 (n=24), A&C 3.5 SE 0.81 (n=19), B&C 4.8 SE 0.39(n=75) and A&B&C 4.1 SE 0.45 (n=61). For the F compartments: FB 1.7 SE 1.14 (n=10), FA 5.0 SE 0.47 (n=63), FI 4.5 SE 1.18 (n=15), FU 7.33 SE 1.54 (n=6). No chronic pelvic pain but with Enzian involvement is present in Supporting Information Table SI n=0 (0.0%) in compartment A, n=38 (30.4%) in compartment B, n=10 (29.4%) in compartment C, n=8 (80.0%) in compartment FB, n=18 (28.6%) in compartment FA, n=6 (40.0%) in compartment FI and n=1 (16.7%) in FU.

### Dyspareunia Figure 1.4 (blue)

A mean pain for compartment A was found of 3.8, SE 1.59 (n=6), B 4.5, SE 0.35 (n=125), C 3.9, SE 0.58 (n=34), A&B 3.8 SE 0.70 (n=24), A&C 3.7 SE 0.82 (n=19), B&C 4.4 SE 0.43 (n=75) and A&B&C 4.6 SE 0.44 (n=61). For the F compartments: FB 4.6 SE 1.09 (n=10), FA 5.7 SE 0.41 (n=63), FI 4.1 SE 1.02 (n=15), FU 6.0 SE 1.29 (n=6). No dyspareunia but with Enzian involvement is present in Supporting Information Table SI n=2 (33.3%) in compartment A, n=41 (32.8%) in compartment B, n=12 (35.3%) in compartment C, n=3 (30.0%) in compartment FB, n=11 (17.5%) in compartment FA, n=6 (40.0%) in compartment FI and n=1 (16.7%) in FU.

### Dysuria Figure 1.5 (yellow)

A mean pain for compartment A was found of 1.4, SE 1.4 (n=6), B 1.7, SE 0.25 (n=125), C 1.9, SE 0.51 (n=34), A&B 2.6 SE 0.68 (n=24), A&C 1.5 SE 0.55 (n=19), B&C 1.7 SE 0.34(n=75) and A&B&C 2.3 SE 0.40 (n=61). For the F compartments: FB 7.1 SE 0.89 (n=10), FA 2.3 SE 0.42 (n=63), FI 2.43 SE 1.00 (n=15), FU 4.2 SE 1.60 (n=6). No dysuria but with Enzian involvement is present in Supporting Information Table SI n=4 (66.7%) in compartment A, n=73 (58.4%) in compartment B, n=22 (64.7%) in compartment C, n=1 (10.0%) in compartment FB, n=35 (55.6%) in compartment FA, n=9 (60.0%) in compartment FI and n=2 (33.3%) in FU.

### ROC curve for ureter and bladder

ROC curves in correlation to the median pain scores were calculated for the Enzian locations A, B, C, FB, FU, FA, and FI. We only present the ROC curves with an area under the curve ≥0.6, since in that case this test can provide a discrimination. This was only the case for the ureter and bladder involvement (Table IV).

**Table IV:** ROC curve Area Under the Curve

Variable(s)	Area	Std. Error^a^	Asymptotic Sig.^b^	Asymptotic 95% Confidence Interval	
				Lower Bound	Upper Bound
Ureter^c^					
dysmenorrhoea 0-10	,521	,038	,588	,446	,595
dysuria 0-10	,516	,039	,681	,439	,592
CPP 0-10	,516	,040	,670	,438	,595
dyspareunia 0-10	,485	,038	,703	,412	,559
dyschezia 0-10	**,601**	,036	,008	,530	,672
Bladder^d^					
dysmenorrhoea 0-10	,486	,039	,728	,409	,563
dysuria 0-10	**,624**	,042	,002	,542	,706
CPP 0-10	,387	,041	,005	,306	,468
dyspareunia 0-10	,440	,039	,133	,364	,516
dyschezia 0-10	,573	,043	,067	,489	,657

^a^Under the nonparametric assumption; ^b^Null hypothesis: true area = 0.5; ^c^71 yes, 304 no, 44 missing (because of no pain reported on some scales); ^d^63 yes, 312 no, 44 missing (because of no pain reported on some scales).

**Figure 2.1:**
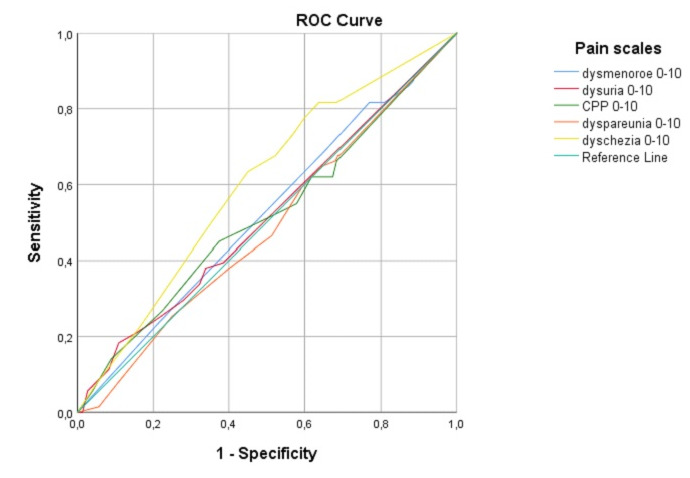
ROC curve of Enzian FU (ureter).

Figure 2.1 shows the ROC curve for the ureter, with an area under the curve for dysmenorrhoea of 0.52 (SE 0.04, CI 0.45-0.60, p=0.04), dysuria 0.51 (SE 0.04, CI 0.44-0.59, p=0.68), CPP 0.52 (SE 0.04, CI 0.44-0.60, p=0.67), dyspareunia 0.49 (SE 0.04, CI 0.41-0.56, p=0.70) and dyschezia 0.6 (SE 0.04, CI 0.53-0.67, p< 0.01) (table IV). We chose the cut- off point for dyschezia of NRS 6.50, while a high sensitivity is important regarding ureter involvement (cave silent hydronephrosis). Using this as a cut-off point, our sensitivity (true positive rate) would be 63% and our 1 – specificity would be 45% (false positive rate).

**Figure 2.2:**
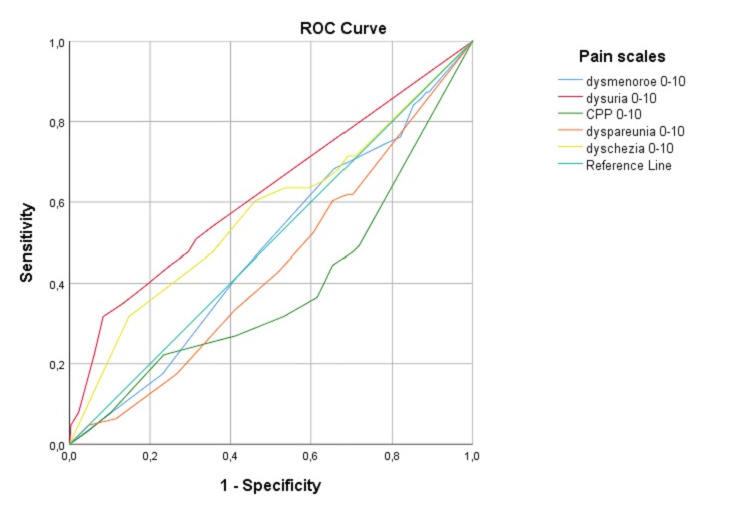
ROC curve of Enzian FB (bladder).

For the bladder (Figure 2.2), with an area under the curve for dysmenorrhoea of 0.49 (SE 0.04, CI 0.41-0.56, p=0.73), dysuria 0.62 (SE 0.04, CI 0.54-0.71, p<0.01), CPP 0.39 (SE 0.04, CI 0.31-0.47, p<0.01), dyspareunia 0.44 (SE 0.04, CI 0.36-0.52, p=0.13) and dyschezia 0.57 (SE 0.04, CI 0.49-0.66, p< 0.07) (table IV). We chose the cut-off point for dysuria of NRS 0.5. Using this as a cut-off point, our sensitivity would be 54% and our 1 – specificity would be 36%.

## Discussion

Preoperative reported pain scores correlate poorly to DE lesions. This underlines and explains the difficulty doctors and patients face, before the diagnosis endometriosis is mae (an average of 9 years) (Pugsley and Ballard, 2007). The correlation of endometriosis involvement to pain symptoms is not a 1-on-1 relationship, this partly explains why undiagnosed patients often see numerous doctors and try several treatment options before the diagnosis of endometriosis is made. This delay makes women lose faith in the healthcare system or they start doubting themselves (Moradi et al., 2014). When the diagnosis is finally made, surgery for removing the endometriosis is not the only solution. These women have often developed chronic pain symptoms which need a broader treatment process than solely removing the physical stimuli (endometriosis nodules). A more holistic approach, which also includes psychological support and pain management/insight is essential to help these women to deal with the consequences of endometriosis and lead a life as normal as possible (Davis-Kankanamge, 2020).

The theory that endometriosis has a multifactorial pathogenesis could explain why endometriosis remains difficult to classify regarding prognosis and clinical correlation. This multifactorial pathogenesis includes endometriosis severity (stage, location, depth of invasion), co-morbidity conditions, myofascial factors (muscle and fascial involvement), and central sensitisation (Yong, 2017). According to the International Association for the Study of Pain, the definition of pain is “an unpleasant sensory and emotional experience associated with actual or potential tissue damage”. Besides, it has become clearer that pain experience is also influenced by psychological factors (e.g., catastrophising, anxiety, coping) (Lame et al., 2005; Laganà et al., 2017) and central sensitisation (persistent state of high reactivity) (Harte et al., 2018). This supports the theory that not only the severity of the endometriosis is responsible for pain symptoms.

Unfortunately, endometriosis invasion of the ureters is often asymptomatic or presents with nonspecific symptoms (Palla et al., 2017), which can lead to silent, obstructive uropathy and renal failure, with a high rate of kidney loss (23–47%) (Charatsi et al., 2018). To detect ureteric involvement, an MRI is the first-choice diagnostic tool (Palla et al., 2017) as it allows proper evaluation of the whole pelvis and helps to decide the surgical approach. In centres with experienced sonographers an ultrasonic approach of detecting endometriosis on the ureters and kidneys would also be an option (Carfagna et al., 2018). In our study we found that dyschezia had a predictive value in detecting ureteric involvement. Translating our research evidence to clinical practice: if endometriosis patients report dyschezia (NRS>6.5), the clinician should be aware of possible ureteric involvement and could extend the transvaginal ultrasound with a MRI (pelvis and kidneys) or at least an ultrasound of the kidneys/ureters. This is in line with the recommendation Koninckx et al. (2012), which stated that hydronephrosis should be excluded in the pre-surgical workup as hydronephrosis is associated with 18% of ureteral lesions.

Most patients with bladder endometriosis are symptomatic (dysuria, voiding problems, etc.) (Abrao et al., 2009; Fauconnier et al., 2002; Knabben et al., 2015). This supports our finding of the symptom of dysuria having a predictive value for the presence of bladder endometriosis and is seen clearly in the euler diagram (Figure 1.5). However, the finding in the ROC curve was not strong, but we can carefully conclude that even low NRS for dysuria (>0.5), could indicate bladder endometriosis. Translated to clinical practice, patients presenting with very little dysuria symptoms can still have bladder endometriosis.

It is interesting to note that adenomyosis (FA) was reported in 58% of all cases, adenomyosis is a radiological finding and can’t be detected intra operatively. Therefore, surgeons must consider that the majority of DE cases also have adenomyosis, and without a hysterectomy symptom of adenomyosis could still remain even though the DE is removed. This could lead to patients who still report pain after the surgery.

Despite the limited correlations, anatomical based endometriosis classification remains important. Classification provides a uniform language to communicate, monitor and report the disease (Jutel, 2011). This enables standardisation of outcome measures and provides a universal way to communicate about endometriosis in clinical and research settings.

An important strength of our study is the fact that this is, to our knowledge, the first study which compared solitary and combinations of anatomical locations in correlation to pain reporting and created ROC curves to determine if certain symptoms could predict the presence of endometriosis in specific compartments. Because of the use of the EQUSUM, data was collected in a uniform way, making it easy to analyse the data without inconsistencies in the datafile.

A limitation of this study is the fact that no medication usage was registered and the NRS pain reporting is pre-operative in patients with and without medication. However, a subgroup analysis within the study from Montanari et al. (2019) showed that preoperative use of hormonal therapy did not influence the associations and correlations for reported pain symptoms described by Enzian. Only a weak correlation was found with the rASRM and dysmenorrhoea when patients used hormonal therapy. So, medication use is obviously of influence, but it seems to have little effect on the results. In future research it would be interesting to not only study the pain intensity, but also the presence or absence of pain and the correlation to the Enzian classification. Pain symptoms rely heavily on subjective perception, particularly the intensity of it (Coghill, 2010). It is known that the absence of pain has much lower interpatient bias compared to the presence of pain (e.g., one patient reports NRS 6, another with the same pain report NRS 8).

A different limitation, but also a strength is the fact that we have small groups in the solitary compartments. Therefore, the statistical strength is weak, however we presented groups without the bias of other compartments. Previous classification studies have greater numbers but do have the bias of other groups (e.g., A3B2C1 would become a group A, by taking the highest group as the main group). Our study chooses not to report the classification that way, which shows more accurate data, but with the limitation of smaller groups.

It has to be noted that in this study design we did not include endometriotic lesions in the ovarian and adhesions scores, which also causes pain. We excluded this in the calculations, otherwise the groups would become too heterogeneous. In future studies it would be interesting to study these effects as well, however this is only possible with larger amounts of cases to keep sufficient cases per group for analysis.

## Conclusion

Our study showed that an increase in pain symptoms does not necessarily goes together with an increase in DE involvement. The symptom dyschezia has some predictive value for ureteric involvement, and therefore patients with dyschezia should preferably get an MRI of the pelvis or an ultrasound scan of the kidneys/ureters for an optimal surgical workup. Minor dysuria symptoms can indicate the presence of endometriosis in the bladder. Increasing evidence suggests that pain symptoms in deep endometriosis has a multifactorial cause, which partly explains the poor correlations in studies focussing solely on anatomical locations and pain symptoms.
